# Comparison of the effects of different types of treatment protocols on the median and overall survival rates of non-small cell lung cancer patients: A real-world retrospective study

**DOI:** 10.1371/journal.pone.0344658

**Published:** 2026-04-09

**Authors:** Tarza Jamal Siahmansur

**Affiliations:** Pharmacology and Toxicology Department, College of Pharmacy, University of Sulaimani, Kurdistan Region Government, Sulaymaniyah, Iraq; Karmanos Cancer Institute, Wayne State University School of Medicine, UNITED STATES OF AMERICA

## Abstract

Lung cancer treatment presents a major challenge globally. The advent of monoclonal antibodies (mAbs) has ushered in a new era in the treatment of non-small cell lung cancer (NSCLC). This study aimed to investigate the impact of the addition of mAbs to platinum-based doublet therapy on overall survival in NSCLC patients and the difference in median survival among patients treated with different treatment protocols. In this study, demographic, clinical, and therapeutic data from 359 NSCLC patients were recorded, and the histopathological and disease stages of the patients, along with the site of metastasis, were documented. The median survival rates, 1-, 3-, and 5-year survival rates, covariate hazard ratios, and independent predictors of overall survival were analyzed. Statistically significant differences were observed in the median survival of patients treated with different treatment protocols (*p =* 0.002). Comparing therapies, patients treated with carboplatin/paclitaxel+anti-PD-1/anti-PD-L1 mAb or cisplatin/vinorelbine+anti-PD-1/anti-PD-L1 mAb had highest median survival (median survival months ± SE) (49.4 months ± 9.15 and 34.9 months ± 8.61 respectively) with lowest hazard ratio (HR = 0.032; 95% confidence interval (CI) [0.003–0.310], *p =* 0.003 and HR = 0.048; 95% CI [0.005–0.465], *p =* 0.0083 respectively). Patients treated with triple therapy [platinum-based doublet chemotherapy combined with mAb drugs had significantly (*p =* 0.01) greater median survival (20.7 months ± 3.11; HR = 0.593; 95% CI [0.443–0.794], *p =* 0.00046) compared to patients treated with platinum-based doublet chemotherapy with concurrent radiation (13.6 months ± 3.8; HR = 0.742 95% CI, [0.531–1.035], *p =* 0.07884] or patients treated with platinum-based doublet chemotherapy (9.1 months ± 0.68; HR = 1) or single treatment (12.9 months ±3.81; HR = 0.927; 95% CI [0.629–1.365], *p =* 0.69970) and better 1-, 3-, and 5-year survival rates (95% CI) [66.20% (0.554–0.77), 26.40% (0.162–0.366), and16.25% (0.065–0.261) respectively] than patients treated with platinum-based doublet chemotherapy with concurrent radiation [54% (0.403–0.677), 22% (0.104–0.336), and 4% (0.001–0.079) respectively] or without concurrent radiation [39.10% (0.324–0.458), 12.80% (0.081–0.175) and 3.57% (0.012–0.381) respectively] or single treatment [51.50% (0.407–0.623), 12.12% (0.0193–0.223), and 10.12% (0.003–0.199) respectively]. The statistically significant predictors for overall survival were cancer metastasis (*p =* 0.0001), disease stage at diagnosis (*p =* 0.001), patient age (*p* = 0.017), and performance of lung resection surgery (*p =* 0.021). 64.1% of the patients had metastasis, and they had significantly lower median survival than patients without metastasis (*p* = 0.0001). Multi-organ metastasis was the most common type of metastasis (20.9%). In conclusion, the addition of mAbs such as anti-programmed cell death protein 1/programmed death-ligand 1 or epidermal growth factor receptor inhibitors or vascular endothelial growth factor inhibitors to platinum-doublet chemotherapy markedly improved the overall survival and survival rates of NSCLC patients.

## Introduction

Lung cancer is one of the most fatal diseases worldwide; it poses a considerable challenge to the world’s public health and is a prominent contributor to cancer-related mortality worldwide. Each year, nearly two million new cases are reported throughout the world, constituting 11.7% of all cancer cases, with 1.8 million associated deaths, accounting for roughly one-fifth of all cancer-related mortalities [1]. This toll surpasses the combined fatalities from breast and colorectal cancers [2]. Lung cancer is a highly metastatic cancer that spreads rapidly to other organs and tissues, particularly to the spinal cord, brain, bone, and liver [3–5].

Lung cancer affects individuals of both sexes and is the most frequently diagnosed cancer in males and the second most common cancer in females [6]. On the basis of its histological characteristics, lung cancer can be broadly categorized into two types: small-cell lung carcinoma (SCLC) and non-small cell lung carcinoma (NSCLC). NSCLC can be further subdivided into adenocarcinoma, squamous cell carcinoma, large cell carcinoma, and undifferentiated carcinoma [7]. NSCLC is more common (85% of cases) than SCLC is, and among NSCLCs, adenocarcinoma is the most common type of NSCLC [8]. According to American Cancer Society data from 2024, the 5-year survival rates for NSCLC and SCLC patients are 28% and 7%, respectively [9].

Management of lung cancer involves surgery, drug therapy, or a combination of both [10–13]. Survival among lung cancer patients became evident between the 1980s and 1990s, after the emergence and adoption of platinum-based chemotherapies (cisplatin, carboplatin, and oxaliplatin) and chemotherapeutic drugs such as Vinka alkaloids (vincristine, vinblastine, vindesine, and vinorelbine), taxanes (paclitaxel, docetaxel, and cabazitaxel), and antimetabolite chemotherapies (gemcitabine, cytarabine, and capecitabine) [14,15]. Platinum chemotherapies are widely used for treating different types of cancer [16–18]. Their work is based on the formation of a protein cross-link that interferes with the repair mechanisms of DNA, leading to DNA damage and thereby causing programmed cell death in cancer cells [19].

Vinca alkaloids are also used for treating NSCLC [20]; they act as microtubule inhibitors by preventing tubulin polymerization, a process essential for spindle formation during the M phase of the cell cycle. At lower concentrations, they impair microtubule function, whereas higher doses lead to cell cycle arrest and apoptosis.

During the early 2000s, pemetrexed (antifolate antineoplastic drug) emerged as an additional modern chemotherapeutic agent that is effective in treating lung cancer [21,22]. Pemetrexed disrupts the activity of at least three key enzymes involved in pyrimidine and purine biosynthesis: thymidylate synthase, dihydrofolate reductase, and glycinamide ribonucleotide formyl transferase [23].

The combination of concurrent radiation with chemotherapy has improved overall survival in lung cancer patients [24–27], even though these types of radiation selectively target tumor cells while minimizing unintended radiation exposure to adjacent healthy tissues [28], unwanted effects on body organs remain [29].

The introduction of monoclonal antibodies (mAbs) marked the beginning of a new era in cancer therapy. The use of antibodies for therapeutic purposes in treating tumors has been a long-standing goal. Therapeutic mAbs bind to their antigens, tumor-associated antigens (TAAs), with high affinity, selectivity, and specificity [30]. They also act as delivery vehicles for carrying cytotoxic drugs, such as radioisotopes and small chemotherapeutic molecules conjugated to cytotoxic agents, to their sites of action [31]. Nowadays, molecular stratification strategies are employed that utilize a patient’s molecular or genetic profile to categorize individuals into distinct subgroups, thereby enabling more personalized therapeutic interventions, enhancing prognostic accuracy, optimizing clinical trial design, and facilitating the discovery of novel therapeutic targets [32–37]

Epidermal growth factor receptor tyrosine kinase (EGFR-TK) signalling is essential for tumor cell propagation, apoptosis prevention, and angiogenesis promotion, and it also facilitates cancer metastasis and reduces the responsiveness of cells to chemotherapy and radiotherapy [38]. To date, there are three generations of EGFR-TKIs; the first generation includes erlotinib, icotinib, and gefitinib, and the second generation includes afatinib and dacomitinib, whereas osimertinib is the only available third generation and is more promising than earlier-generation EGFR-TKIs [39]. EGFR-TKI drugs are good choices for NSCLC patients with a mutation in the epidermal growth factor receptor gene. In several studies, researchers reported that individuals who received EGFR-TK targeted therapy had significantly better progression-free survival (PFS) than patients who received platinum-based therapies did, whereas overall survival (OS) increased significantly in only a few of them [39–43].

Bevacizumab is a genetically engineered humanised immunoglobulin 1 antibody that is directed against vascular endothelial growth factor (VEGF). VEGF is critically involved in angiogenesis, and its overproduction is linked to tumor progression and a worse disease prognosis [44]. In an experimental study, Masuda et al*.* confirmed that the use of bevacizumab in models with VEGF mutations results in improved antitumour activity [45]. In addition, in a clinical trial, Zhou et al. revealed that patients with advanced-stage NSCLC who were treated with chemotherapy combined with bevacizumab experienced progression-free survival that was 3–7 months longer than that of patients treated with erlotinib alone [46].

Nivolumab is the first programmed cell death protein 1 (PD-1) PD-1 inhibitor mAb used as a treatment for NSCLC cases, which considerably enhances the objective remission rate and overall survival in comparison with platinum-based chemotherapy [47]. PD-1 inhibitors function by blocking the interaction between the PD-1 receptor and its ligands, PD-L1 and PD-L2. This blockade removes the immune system’s suppression, enabling T cells to identify and destroy cancer cells, which may result in tumor regression or a slowdown in tumor progression for certain patients [48]. In a randomized open-label, phase III clinical trial of 352 resectable NSCLC patients with stages IB to IIIA disease (equally allocated into the two treatment groups), Forde P.M. et al revealed that the median event-free survival was 31.6 months (95% CI: 30.2 to not reached) in the nivolumab plus chemotherapy group compared with 20.8 months (95% CI: 14.0–26.7) in the chemotherapy-only group, and the hazard ratio for disease progression, recurrence, or death was 0.63 (97.38% CI: 0.43–0.91), indicating a statistically significant difference (*p* = 0.005) [49]. However, in the CheckMate 331 clinical trial, nivolumab did not significantly improve OS compared with chemotherapy-treated patients with relapsed SCLC [50]. Atezolizumab and pembrolizumab (humanised IgG4 anti-PD-1 mAb) are effective treatment regimens for metastatic NSCLC [51]. Compared with conventional therapy, both methods result in better overall survival and progression-free survival in NSCLC patients [52–55]. In the OAK study (a randomized, open-label phase III clinical trial carried out at 194 academic and community-based practice centers across 31 nations), treatment with atezolizumab resulted in improved overall survival compared with docetaxel, with a median overall survival of 13.8 months (95% CI: 11.8–15.7) versus 9.6 months (95% CI: 8.6–11.2). The hazard ratio was 0.73 (95% CI: 0.62–0.87), indicating a statistically significant benefit (*p* = 0.0003) [54] Furthermore, in the KEYNOTE-189 study, which compared the effects of the addition of either pembrolizumab or placebo to platinum-pemetrexed therapy, Garassino M.C. et al. reported that the five-year OS rates in both groups were 19.4% in the pembrolizumab group and 11.3% in the placebo group [53]. However, in the EYNOTE-604 randomized phase III clinical study of 453 patients with SCLC, the patients were assigned to two arms: etoposide and platinum (EP) with a placebo plus etoposide and platinum to compare the effects of the drugs on PFS and OS, and the combination of pembrolizumab with EP resulted in a significant improvement in progression-free survival (PFS), with a hazard ratio of 0.75 (95% CI: 0.61–0.91; *p* = 0.0023). At 12 months, the PFS was 13.6% in the pembrolizumab plus EP arm and 3.1% in the placebo plus EP arm. Overall survival (OS) was prolonged with pembrolizumab plus EP, but the difference was not statistically significant (HR = 0.80, 95% CI: 0.64–0.98, *p* = 0.0164). The two-year OS rates were 22.5% and 11.2%, respectively [56]

These controversies about the best treatment strategies for patients with lung cancer highlight the need for further investigations. Although selecting appropriate treatment protocols for patients with lung cancer is crucial for improving patient survival rates, studies addressing this issue are lacking. This study aims to investigate the effects of various therapeutic strategies on median and overall survival in individuals diagnosed with NSCLC and the impact of the addition of mAbs to chemotherapies to identify the strongest predictors of overall survival in NSCLC patients and the most common type of metastasis, thereby providing additional insights to complement earlier studies. The null hypothesis of this study posits that there are no statistically significant differences in overall survival rates among NSCLC patients receiving different treatment strategies.

## Methods

### Study design

This research was carried out as a retrospective study after obtaining ethical approval for the study from the Ethics Committee of the College of Pharmacy at the University of Sulaimani (Registration number PH122-24, dated March 26, 2024). The study population included patients diagnosed with NSCLC who visited the Medical Oncology Department at Hiwa Cancer Hospital, Sulaymaniyah, Iraq, between January 1, 2016, and December 31, 2022. A total of 547 patients were admitted and diagnosed with NSCLC during that period, from that 359 patients were eligible to be included in this study who had the same ethnic background completed their treatment entirely at Hiwa Hospital, had complete demographic, medical and therapeutic records, and died from lung cancer. Data collection was conducted from July 10, 2024, to December 1, 2024, and participants’ names were anonymized prior to analysis.

### Data collection

This study recorded the demographic and clinical data of the patients, including age, sex, ethnicity, BMI, smoking status, comorbidities, having family members with lung cancer, and treatment protocols used to treat the patients. In addition, the date of diagnosis and survival duration were recorded, as were whether lung resection surgery had been performed. The histopathological subtype and disease stage were also documented, along with the site of metastasis, if present.

### Statistical analysis

The data were statistically analyzed via SPSS software (version 25.0; SPSS Inc., Chicago, IL) and Python programming language version 3.10, developed by The Python Software Foundation, located in Wilmington, DE, USA. A P value of 0.05 or less was considered statistically significant. The median survival rates and 1-, 3- and 5-year survival rates were evaluated via Kaplan–Meier survival curves. Group differences were evaluated with the log-rank test. A Cox proportional hazards (CPH) model was employed to analyze time-to-event hazard ratios, with the proportional hazards assumption evaluated using Schoenfeld residuals. To identify independent predictors of overall survival, the Cox proportional hazards regression model was applied. Patients were right-censored if the event had not occurred within the defined time window. The backwards LR selection method was used in the multivariate model to determine the strongest predictors of disease outcomes.

## Results

### Demographic and clinical parameters with median survival

This study included three hundred fifty-nine NSCLC patients (280 males and 79 females), with a mean age of 66 years (range: 30–89 years) (Table 1). Most of the patients had a normal BMI (49.3%). Adenocarcinoma was the most common type (47.9%), followed by squamous cell carcinoma (40.9%), whereas large cell carcinoma was the least common type (1.7%). The majority of the patients were diagnosed at stage 4 (62.4%) or stage 3 (24.8%); however, only a few patients were diagnosed at stage 2 (10%) or stage 1 (2.8%). A small number of patients underwent lung resection surgery (19.8%). The median survival ± standard error (SE) for all of the patients was 11.2 months ± 0.83, and 12.8% of the total patients were censored.

**Table 1:** Demographic and clinical data of the patients and median survival (Months ± SE), with percentages censored.

Variables	Groups	N (%)	Median survival (Months) ± SE	Percent censored	P value
**Sex**	Male	280 (78%)	10.3 ± 0.77	15.2%	0.095
	Female	79 (22%)	17.2 ± 3.03	12.1%	
**Age group (years)**	<50	26 (7.2%)	23.4 ± 6.62	23.1%	0.03*
	50-59	77 (21.4%)	11.3 ± 2.67	16.9%	
	60-69	127 (35.4%)	10.3 ± 1.18	8.7%	
	70-79	113 (31.5%)	11.8 ± 1.3	14.2%	
	>80	16 (4.5%)	10.7 ± 2.9	0%	
**BMI (kg/m**^**2**^)	Underweight	50 (13.9%)	10.1 ± 1.94	4%	0.074
	Normal	177 (49.3%)	12.9 ± 1.55	11.9%	
	Overweight	91 (25.3%)	11.2 ± 1.37	14.3%	
	Obese	41 (11.5%)	13.2 ± 7.00	24.4%	
**NSCLC types**	adenocarcinoma	172 (47.9%)	12.7 ± 1.86	12.2%	0.059
	squamous cell	147 (40.9%)	12.4 ± 1.30	14.3%	
	undifferentiated	34 (9.5%)	8.2 ± 1.20	11.8%	
	large cell	6 (1.7%)	3.9 ± 0.74	0%	
**Stage at diagnosis**	1	10 (2.8%)	54.3 ± 42.79	50%	0.0001****
	2	36 (10%)	24.4 ± 1.86	30.6%	
	3	89 (24.8%)	14.8 ± 3.27	12.4%	
	4	224 (62.4%)	8.9 ± 0.76	8.5%	
**Lung resection** **surgery**	Yes	71 (19.8%)	16.8 ± 3.38	25.4%	0.0001****
	No	288 (80.2%)	10.5 ± 0.71	9.7%	
**Smoking**	Yes	239 (66.6%)	10.5 ± 0.64	12.1%	0.135
	No	120 (33.4%)	15.8 ± 1.98	14.2%	
**Family members with lung cancer**	Yes	61 (17%)	8.9 ± 1.14	12.1%	0.509
	No	298 (83%)	12.7 ± 0.95	14.2%	
**Chronic Diseases**	Diabetes	23 (6.4%)	9.7 ± 1.99	8.7%	0.226
	Heart diseases	3 (0.8%)	9.5 ± 0.43	0%	
	Hypertension	23 (6.4%)	12.8 ± 6.30	8.7%	
	Multi-morbidity	16 (4.5%)	16.6 ± 20.09	31.3%	
	No	294 (81.8%)	11.3 ± 0.97	12.6%	
**Metastasis**	Yes	230 (64.1%)	9.5 ± 0.628	6.5%	0.0001****
	No	129 (35.9%)	19.1 ± 3.04	24%	
**Total**		359 (100%)	11.2 ± 0.83	12.8%	

Descriptive analysis was performed via SPSS; the data are presented as the total number of patients (percentages). *p < 0.05, **p < 0.01, and ***p < 0.001, and ****p < 0.0001.

Kaplan–Meier survival analysis was performed via SPSS. The data are presented as the median survival (median ± SE), and the percentages of cases were censored.

The log-rank test was employed to examine group differences.

A greater percentage of patients were smokers (66.6%). Seventeen percent of the patients had at least one family member diagnosed with lung cancer. Hypertension and diabetes were the most common chronic diseases (6.4% each). Most of the patients (64.1%) had metastasis of lung cancer to one or multiple body organs or tissues.

The median survival rates were significantly greater for patients who were diagnosed earlier (p = 0.0001), patients who underwent lung cancer tumor removal surgery (p = 0.0001), patients with no tumor metastasis (p = 0.0001), and younger patients (p = 0.03). Patients diagnosed with adenocarcinoma and squamous cell carcinoma had longer median survival than undifferentiated and large cell carcinoma patients did, but the difference was not significant (p = 0.059).

### Comparison of the effects of different treatment protocols on median survival

**Fig 1:**
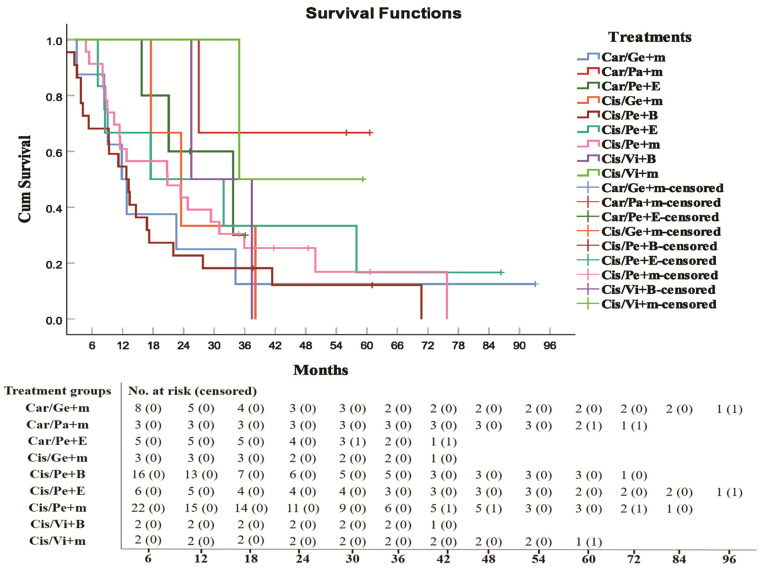
Survival analysis and number at risk of triple therapy in NSCLC patients. Carboplatin/Gemcitabine+anti-PD-1/anti-PD-L1 mAb (Car/Ge+m), Carboplatin/Paclitaxel+anti-PD-1/anti-PD-L1 mAb (Car/Pa+m), Carboplatin/Pemetrexed+Erlotinib (Car/Pe+E), Cisplatin/Gemcitabine+anti-PD-1/anti-PD-L1 mAb (Cis/Ge+m), Cisplatin/Pemetrexed+Bevacizumab (Cis/Pe+B), Cisplatin/Pemetrexed+Erlotinib (Cis/Pe+E), Cisplatin/Pemetrexed+anti-PD-1/anti-PD-L1 mAb (Cis/Pe+m), Cisplatin/Vinorelbine+Bevacizumab (Cis/Vi+B), Cisplatin/Vinorelbine+anti-PD-1/anti-PD-L1 mAb (Cis/Vi+m).

**Fig 2:**
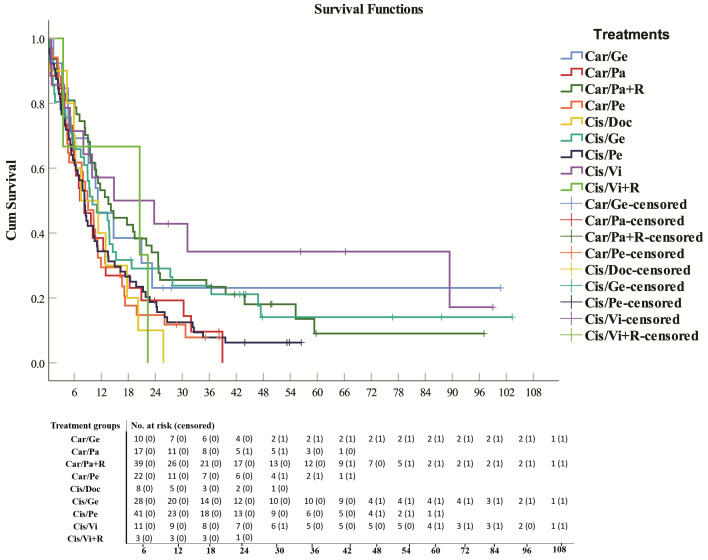
Survival analysis and number at risk of platinum-based doublet chemotherapy with or without concurrent radiation in NSCLC patients. Carboplatin/Gemcitabine (Car/Ge), Carboplatin/Paclitaxel (Car/Pa), Carboplatin/Paclitaxel with concurrent Radiation (Car/Pa+R), Carboplatin/Pemetrexed (Car/Pe), Cisplatin/Docetaxel (Cis/Doc), Cisplatin/Gemcitabine (Cis/Ge), Cisplatin/Pemetrexed (Cis/Pe), Cisplatin/Vinorelbine (Cis/Vi), Cisplatin/Vinorelbine with concurrent Radiation (Cis/Vi+R).

**Fig 3:**
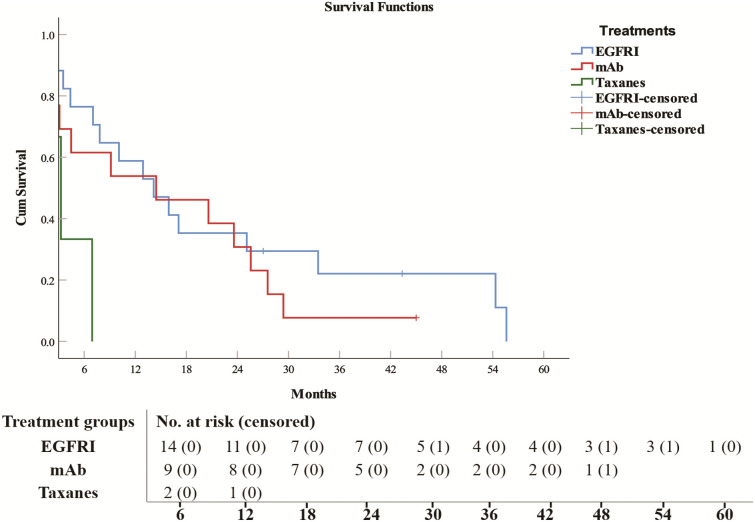
Survival analysis and number at risk of single therapy in NSCLC patients. Epidermal growth factor inhibitors (EGFRI), anti-programmed death 1 (anti-PD-1)/anti-programmed death ligand 1 monoclonal antibodies (mAb).

**Fig 4:**
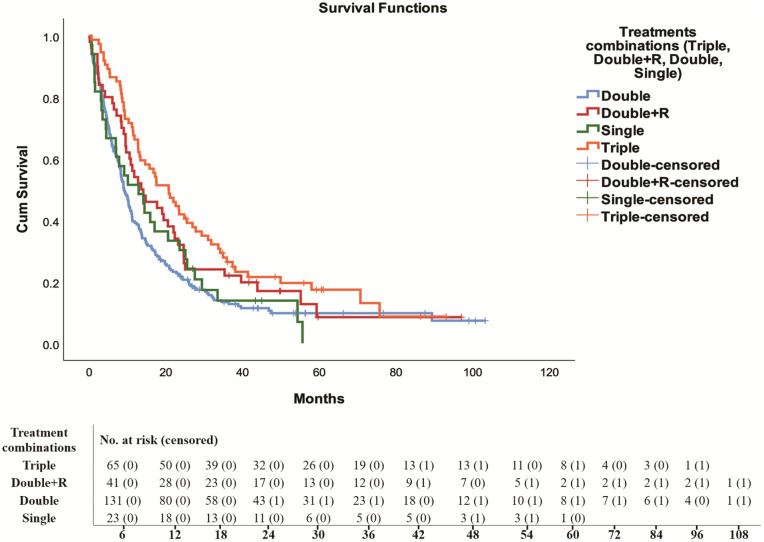
Survival analysis and number at risk of triple therapy, platinum-based doublet chemotherapy with or without concurrent radiation and single drug treatment in NSCLC patients.

There were highly statistically significant differences in median survival among patients treated with different treatment protocols (*p =* 0.002) (Table 2) (Figs 1–3). Patients treated with triple therapy: platinum-based doublet therapy (an alkylating agent combined with either taxanes, vinca alkaloids, folate antimetabolites or antimicrotubular agents) followed by either anti-PD-1/anti-PD-L1 mAb or EGFRI or VEGFI had significantly (*p* = 0.01) greater median survival and more censored patients with lowest hazard ratio (median survival months ± SE) (20.7 months ± 3.11, HR = 0.593, 95% CI: 0.443–0.794, *p* = 0.00046) than patients receivied platinum-based doublet therapy with or without radiation or single treatment (13.6 months ± 3.8, HR = 0.742, 95% CI: 0.531–1.035, *p* = 0.07884), (9.1 ± 0.68, HR = 1), and (12.9 months ± 3.81, HR = 0.927, 95% CI: 0.629–1.365, *p* = 0.6997) respectively (Table 3) (Fig 4).

**Table 2:** Comparison of the effects of different treatment protocols on median survival in lung cancer patients.

Treatments	N (%)	Median Survival (Months) ± SE	Percent censored	HR (95% CI)	P value
Carboplatin/Paclitaxel+anti-PD-1/anti-PD-L1 mAb	5 (1.4%)	49.4 ± 9.15	66.7%	0.032 (0.003-0.310)	0.003**
Cisplatin/Vinorelbine+anti-PD-1/anti-PD-L1 mAb	2 (0.6%)	34.9 ± 8.61	50%	0.048 (0.005-0.465)	0.0083**
Carboplatin/Pemetrexed+Erlotinib	5 (1.4%)	33.7 ± 10.07	40%	0.086 (0.017-0.429)	0.0028**
Cisplatin/Vinorelbine+Bevacizumab	2 (0.6%)	25.5 ± 5.96	0%	0.123 (0.020-0.742)	0.022*
Cisplatin/Gemcitabine+anti-PD-1/anti-PD-L1 mAb	3 (0.8%)	23.5 ± 4.85	0%	0.144 (0.029-0.722)	0.0183*
Cisplatin/Pemetrexed+anti-PD-1/anti-PD-L1 mAb	23 (6.4%)	20.8 ± 8.47	17.4%	0.135 (0.039-0.462)	0.0014
Cisplatin/Pemetrexed+Erlotinib	6 (1.7%)	17.5 ± 14.28	16.7%	0.116 (0.027-0.494)	0.0035**
Cisplatin/Pemetrexed+Bevacizumab	22 (6.1%)	12.7 ± 2.37	9.1%	0.203 (0.060-0.693)	0.0107*
Carboplatin/Gemcitabine+anti-PD-1/anti-PD-L1 mAb	8 (2.2%)	11.8 ± 2.65	12.5%	0.165 (0.042-0.647)	0.0096**
Cisplatin/Vinorelbine with sequential Radiation	3 (0.8%)	20.5 ± 13.89	0%	0.242 (0.048-1.212)	0.0832
Carboplatin/Paclitaxel with concurrent Radiation	47 (13.1%)	13.6 ± 2.36	14.9%	0.167 (0.051-0.548)	0.0031**
Cisplatin/Vinorelbine	14 (3.9%)	14.8 ± 12.91	28.6%	0.114 (0.031-0.423)	0.0011**
Carboplatin/Gemcitabine	13 (3.6%)	11.2 ± 3.35	23.1%	0.171 (0.047-0.630)	0.0078**
Cisplatin/Gemcitabine	41 (11.4%)	10.1 ± 2.82	17.1%	0.185 (0.056-0.611)	0.0055**
Carboplatin/Pemetrexed	34 (9.5%)	8.9 ± 1.70	8.8%	0.287 (0.087-0.949)	0.0402*
Cisplatin/Pemetrexed	64 (17.8%)	8.3 ± 0.79	6.3%	0.276 (0.086-0.890)	0.0306*
Cisplatin/Docetaxel	10 (2.8%)	7.5 ± 4.00	0%	0.319 (0.087-1.168)	0.0835
Carboplatin/Paclitaxel	26 (7.2%)	7.3 ± 2.11	7.7%	0.282 (0.084-0.945)	0.0395*
anti-PD-1/anti-PD-L1 mAb [Atezolizumab, Nivolumab, Pembrolizumab (mAb)	13 (3.6%)	14.5 ± 9.66	7.7%	0.222 (0.062-0.797)	0.0207*
Alectinib, Erlotinib, Gefitinib, Osimertinib (EGFRI)	17 (4.7%)	14.2 ± 4.01	11.8%	0.182 (0.052-0.637)	0.0076**
Taxane (Paclitaxel or Docetaxel)	3 (0.8%)	3.3 ± 1.39	0%	1	
Overall	359 (100%)	11.2 ± 0.83	12.8%		

Descriptive analysis was performed via SPSS; the data are presented as the total number of patients (percent). *p < 0.05, **p < 0.01, and ***p < 0.001, and ****p < 0.0001.

Kaplan–Meier survival analysis was performed via SPSS. The data are presented as the median survival (median ± SE), and the percentages of cases were censored.

The log-rank test was employed to examine group differences.

**Table 3:** Comparison of the effects of triple therapy, platinum-based doublet chemotherapy with or without concurrent radiation, and single drug treatment on median survival in lung cancer patients.

Drugs	N (%)	Median Survival (Months) ± SE	Percent censored	HR (95% CI)	P value
**Triple therapy**	81 (22.6%)	20.7 ± 3.11	16.0%	0.593 (0.443-0.794)	0.00046 ***
**Platinum-based doublet chemotherapy plus radiation**	50 (13.9%)	13.6 ± 3.8	14.0%	0.742 (0.531-1.035)	0.07884
**Platinum-based doublet chemotherapy**	195 (54.3%)	9.1 ± 0.68	11.8%	1	
**Single therapy**	33 (9.2%)	12.9 ± 3.81	9.1%	0.927 (0.629-1.365)	0.69970
**Total**	359 (100%)	11.2 ± 0.83	12.8%		

Descriptive analysis was performed via SPSS; the data are presented as the total number of patients (percentages). *p < 0.05, **p < 0.01, and ***p < 0.001, and ****p < 0.0001.

Kaplan–Meier survival analysis was performed via SPSS. The data are presented as the median survival (median ± SE), and the percentages of cases were censored.

The log-rank test was used to examine group differences.

Among triple therapies, carboplatin/paclitaxel plus anti-PD-1/anti-PD-L1 mAb (median survival months ± SE) (49.4 months ± 9.15, HR = 0.032, 95% CI: 0.003–0.310, *p* = 0.003), cisplatin/vinorelbine plus anti-PD-1/anti-PD-L1 mAb (34.9 months ± 8.61; HR = 0.048, 95% CI: 0.005–0.465, *p* = 0.0083), and carboplatin/pemetrexed plus erlotinib (33.7 months ± 10.07, HR = 0.086, 95% CI: 0.017–0.429, *p* = 0.0028) had higher median survival rates and lowest hazard ratio than did other triple therapies.

The median survival was significantly (*p* = 0.05) higher in patients who received concurrent radiation therapy with their chemotherapy treatment (carboplatin/paclitaxel+concurrent radiation) (13.6 months ± 2.36; HR = 0.167, 95% CI: 0.051–0.548, *p* = 0.0031) than in patients who received carboplatin/paclitaxel alone (7.3 months ± 2.11, HR = 0.282, 95% CI: 0.084–0.945, *p* = 0.0395). In addition, patients treated with concurrent radiation therapy accompanied by cisplatin/vinorelbine therapy had greater median survival but not significantly (*p* = 0.3) (20.5 months ± 13.89, HR = 0.242, 95% CI: 0.048–1.212, *p* = 0.0832) than patients treated with cisplatin/vinorelbine alone (14.8 months ±12.91, HR = 0.114, 95% CI: 0.031–0.423, *p* = 0.0011).

Among platinum-based doublet therapies, cisplatin/vinorelbine (14.8 months ± 12.91, HR = 0.114, 95% CI: 0.031–0.423, *p* = 0.0011) was superior to other drug combinations in terms of survival, whereas for single-agent treatment, anti-PD-1/anti-PD-L1 mAbs and EGFRIs had similar survival periods (14.5 months ± 9.66, HR = 0.222, 95% CI: 0.062–0.797, *p* = 0.0207) and (14.2 months ± 4.01, HR = 0.182, 95% CI: 0.052–0.637, *p* = 0.0076) respectively, and they were remarkably longer than those of taxane drugs (paclitaxel and docetaxel) (3.3 months ± 1.39, HR = 1).

### 1-year, 3-year, and 5-year survival rates

Compared with platinum-based doublet chemotherapy with or without concurrent radiation and single drug therapy, triple therapy had the highest survival rates in terms of all 1-year, 3-year, and 5-year survival rates, while the lowest survival rate was for platinum-based doublet chemotherapy (Table 4).

**Table 4:** Comparison of 1-year, 3-year, and 5-year survival rates between triple therapy, platinum-based doublet chemotherapy with or without concurrent radiation, and single-drug treatment.

Survival rates	Triple therapy [Percent (95% CI)]	Platinum-based doublet chemotherapy +Radiation [Percent (95% CI)]	Platinum-based doublet chemotherapy [Percent (95% CI)]	Single therapy [Percent (95% CI)]
**1 year**	66.2% (0.554-0.77)	54% (0.403-0.677)	39.1% (0.324-0.458)	51.5% (0.407-0.623)
**3 years**	26.4% (0.162-0.366)	22% (0.104-0.336)	12.8% (0.081-0.175)	12.12% (0.0193-0.223)
**5 years**	16.25% (0.065-0.261)	4% (0.001-0.079)	3.57% (0.012-0.381)	10.12% (0.003-0.199)

### Hazard ratio and covariate effects on overall survival

**Fig 5:**
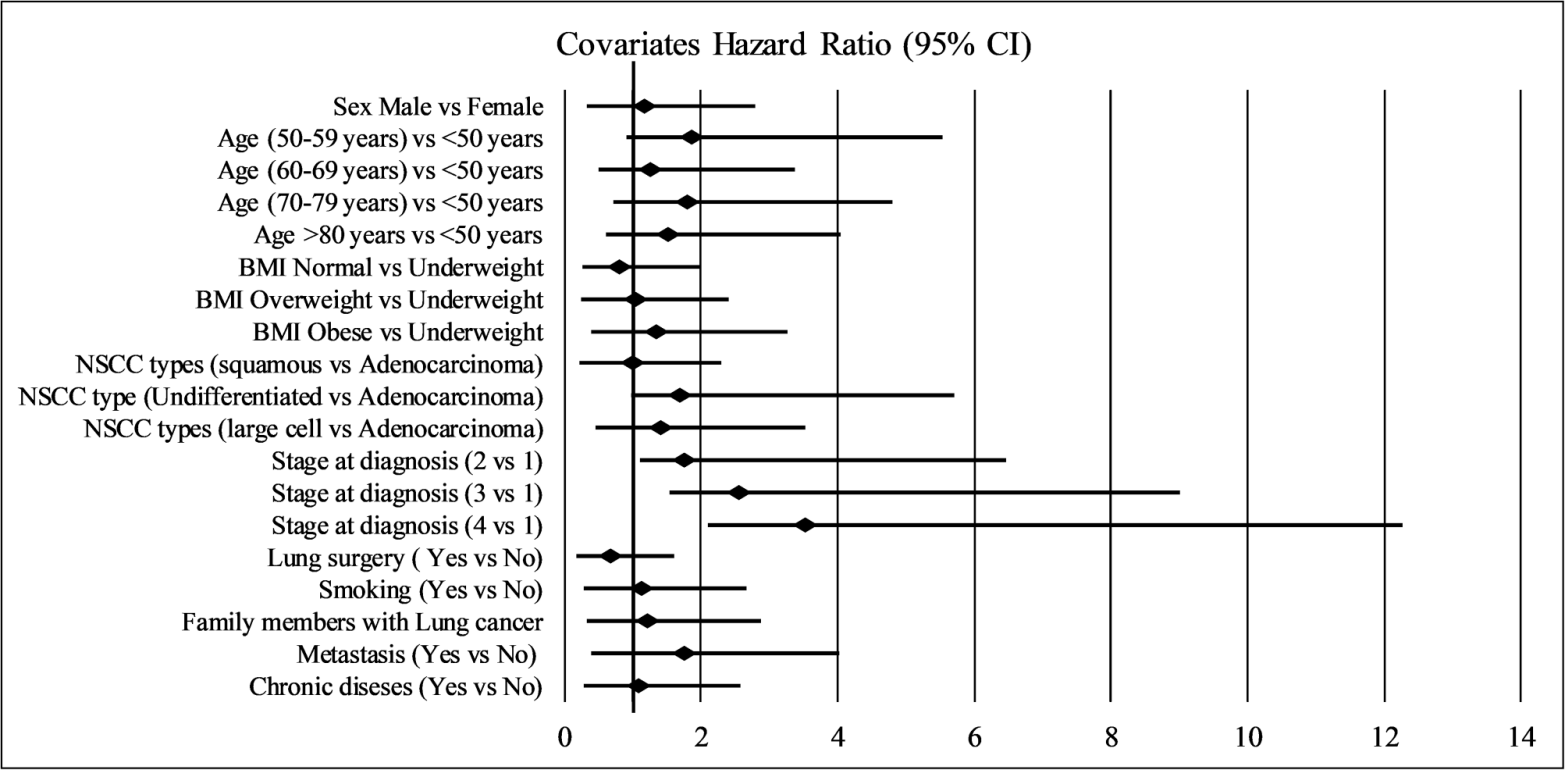
Covariates hazard ratio (95% CI) using Cox regression analysis.

In this study, the proportional hazards assumption was held as shown in Table 5. Cox regression analysis was used to analyse the hazard ratios of the covariates (Table 5) (Fig 5). According to the results, the hazard ratio was greater for males than for females for underweight and obese patients than for normal weight patients for patients recognized with undifferentiated carcinoma and large cell carcinoma than for patients recognized with adenocarcinoma and sequential cell carcinoma, for smoking patients, for patients with chronic disease, and for those who had one or more family members recognized with lung

**Table 5:** Covariates proportional hazards assumption and hazard ratio.

	CPH (P value)	HR (95% CI)	
Sex (Male vs Female)	0.108	1.178 (0.853-1.626)	
Age group (Years)	0.384		
Age (50–59 years) vs <50 years		1.868 (0.949-3.674)	
Age (60–69 years) vs <50 years		1.255 (0.740-2.130)	
Age (70–79 years) vs <50 years		1.805 (1.088-2.995)	
Age >80 years vs <50 years		1.522 (0.916-2.528)	
BMI (kg/m^2^)	0.325		
BMI Normal vs Underweight		0.800 (0.538-1.189)	
BMI Overweight vs Underweight		1.037 (0.783-1.373)	
BMI Obese vs Underweight		1.352 (0.957-1.910)	
NSCC types	0.085		
NSCC types (squamous vs Adenocarcinoma)		0.997 (0.763-1.303)	
NSCC type (Undifferentiated vs Adenocarcinoma)		1.695 (0.716-4.013)	
NSCC types (large cell vs Adenocarcinoma)		1.411 (0.944-2.108)	
Stage at diagnosis	0.06		
Stage at diagnosis (2 vs 1)		1.762 (0.661-4.698)	
Stage at diagnosis (3 vs 1)		2.561 (1.017-6.450)	
Stage at diagnosis (4 vs 1)		3.524 (1.420-8.746)	
Lung resection surgery (Yes vs No)	0.393	0.673 (0.489-0.926)	
Smoking (Yes vs No)	0.29	1.125 (0.825-1.533)	
Family members with lung cancer	0.18	1.213 (0.885-1.664)	
Metastasis (Yes vs No)	0.141	1.756 (1.364-2.261)	
Chronic diseases (Yes vs No)	0.963	1.094 (0.808-1.481)	

While using the backwards LR selection method in multivariate analysis, cancer metastasis, stage at diagnosis, patient status, and lung resection surgery were statistically significant predictors of overall survival (Table 6).

**Table 6:** Covariate effects on overall survival.

Covariates	Groups	HR (95% CI)	P value
**Age (Years) VS <50**	50-59	1.949 (0.994-3.819)	0.017*
	60-69	1.294 (0.772-2.167)	
	70-79	1.967 (1.207-3.204)	
	>80	1.659 (1.015-2.71)	
**Stage at diagnosis VS stage 1**	Stage 2	1.76 (0.663-4.677)	0.001***
	Stage 3	2.546 (1.012-6.405)	
	Stage 4	3.414 (1.38-8.447)	
**Lung Surgery performed**	Yes Vs No	0.691 (0.505-0.946)	0.021*
**Metastasis**	Yes Vs No	1.786 (1.393-2.291)	0.0001****

Multivariate analysis was performed via the backwards LR selection method. *p < 0.05, **p < 0.01, ***p < 0.001, and ****p < 0.0001.

### Metastasis and overall survival

**Fig 6:**
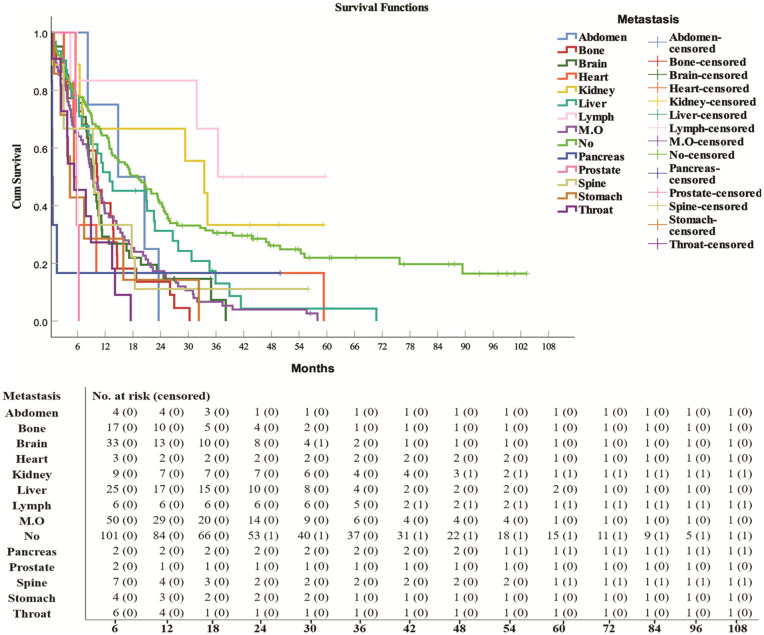
The overall survival and number at risk of patients with different organ metastasis. (M.O) multi organ metastasis, (No) no metastasis.

The results revealed that more than sixty percent of the patients had metastasis (64.1%) (Table 1). Patients with metastasis had significantly lower median survival than patients without metastasis (p = 0.0001) (Table 1). The median survival was significantly different between patients with different types of disease metastasis (p = 0.0001) (Table 6). (Table 7) (Fig 6). The most common type of metastasis was multiorgan metastasis (20.9%), followed by brain (11.4%), liver (8.6%), and bone (6.1%) metastasis. Compared with patients with other organ metastasis, patients with lymph node metastasis (36.4 months ± 8.31, HR = 0.454, 95% CI: 0.144–1.434, p = 0.1782), kidney (33.5 months ± 6.17, HR = 0.73, 95% CI: 0.32–1.666, p = 0.4543), abdominal (14.8 months ± 6.14; HR = 1.708, 95% CI: 0.626–4.661, p = 0.2961), and liver (13 months ±5.82, HR = 1.56, 95% CI: 1.028–2.367, p = 0.0367) metastasis survived better. A lower median survival was observed for patients with pancreas metastasis (0.5 months ±0.08; HR = 3.278, 95% CI: 1.330–8.081, p = 0.0099), stomach (4.4 months ±0.77, HR = 3.069, 95% CI: 1.42–6.637, p = 0.0044), heart (5.3 months ±0.46, HR = 2.291, 95% CI: 1.001–5.240, p = 0.0496), throat (5.3 months ±2.22, HR = 3.936, 95% CI: 2.086–7.424, p = 0.00001), and prostate (5.9 months ±0.32, HR = 5.113, 95% CI: 1.596–16.382, p = 0.006) metastasis.

**Table 7:** Metastasis of lung cancer to body organs and median survival of patients with different types of organ metastasis.

Metastasis organs	N (%)	Median Survival months ± SE	Percent censored	Hazard ratio (95% CI)	P value
**Lymph node**	6 (1.7%)	36.4 ± 8.31	50%	0.454 (0.144-1.434)	0.1782
**Kidney**	9 (2.5%)	33.5 ± 6.17	33.3%	0.73 (0.32-1.666)	0.4543
**Abdomen**	4 (1.1%)	14.8 ± 6.14	0%	1.708 (0.626-4.661)	0.2961
**Liver**	31 (8.6%)	13.0 ± 5.82	6.5%	1.56 (1.028-2.367)	0.0367*
**Bone**	22 (6.1%)	10.2 ± 1.68	0%	2.43 (1.517-3.893)	0.0002***
**Spleen**	9 (2.5%)	9.7 ± 0.49	11.1%	1.954 (0.948-4.029)	0.0694
**Brain**	41 (11.4%)	9.5 ± 0.76	9.8%	2.110 (1.433-3.106)	0.0002***
**Multi organs**	75 (20.9%)	9.1 ± 1.17	1.3%	2.193 (1.611-2.984)	0.00001****
**Prostate**	3 (0.8%)	5.9 ± 0.32	0%	5.113 (1.596-16.382)	0.006**
**Throat**	11 (3.1%)	5.3 ± 2.22	0%	3.936 (2.086-7.424)	0.00001****
**Heart**	6 (1.7%)	5.3 ± 0.46	0%	2.291 (1.001-5.240)	0.0496*
**Stomach**	7 (1.9%)	4.4 ± 0.77	0%	3.069 (1.42-6.637)	0.0044**
**Pancreas**	6 (1.7%)	0.5 ± 0.08	16.7%	3.278 (1.330-8.081)	0.0099**
**No Metastasis**	129 (35.9%)	19.1 ± 3.04	24%	1	

Descriptive analysis was performed via SPSS; the data are presented as the total number of patients (percentages). *p < 0.05, **p < 0.01, and ***p < 0.001, and ****p < 0.0001.

Kaplan–Meier survival analysis was performed via SPSS. The data are presented as the median survival (median ± SE), and the percentages of cases were censored.

The log-rank test was used to examine group differences.

## Discussion

In this study, data of 359 NSCLC patients were analyzed who were assigned to different treatment protocols based on disease stage, overall clinical status, comorbidities, and available molecular profiling results. Results highlighted the effects of different treatment protocols used to treat patients with NSCLC on median survival and overall survival rates, and the enhancing effect of the addition of mAb treatment to platinum-based doublet chemotherapy. The results revealed that the addition of either anti-PD-1/anti-PD-L1 monoclonal antibodies (atezolizumab, nivolumab, or pembrolizumab), EGFRI (alectinib, erlotinib, gefitinib, or osimertinib), or VEGFI (bevacizumab) to platinum-based doublet chemotherapy (cisplatin or carboplatin combined with one of the following treatments: vinorelbine, paclitaxel, pemetrexed, or gemcitabine) significantly improved the median survival and survival rates of patients at all disease stages. Among these patients, those receiving carboplatin/paclitaxel + anti-PD-1/anti-PD-L1 mAb (49.4 months ± 9.15,HR=0.032, 95% CI: 0.003-0.310, *p*=0.003), cisplatin/vinorelbine+anti-PD-1/anti-PD-L1 mAb (34.9 months±8.61, HR=0.048, 95% CI: 0.005-0.465, *p*=0.0083), and carboplatin/pemetrexed+erlotinib (33.7 months±10.07, HR=0.086, 95% CI: 0.017-0.429, *p*=0.0028) had greater median survival and lowest hazard ratio than did those receiving other triple therapies.

Evidence from the ECOG 4599 American trial, which enrolled 878 patients with stage IIIB or IV NSCLC, supports these findings, as participants were randomly assigned to receive either paclitaxel and carboplatin alone or in combination with bevacizumab. The addition of bevacizumab significantly improved outcomes, with overall survival increasing to 12.3 months compared with 10.3 months (*p=*0.003) and progression-free survival increasing to 6.2 months versus 4.5 months (*p*<0.001) [57]. In another clinical study, Gandhi L. et al*.* reported that combining pembrolizumab with chemotherapy markedly improved OS compared with patients who received chemotherapy alone [52]. Additionally, in a randomized, multicenter, double-blind phase III clinical study of 550 patients, researchers revealed that the addition of nivolumab to either the carboplatin, paclitaxel, or bevacizumab regimen significantly improved PFS (12.1 vs. 8.1 months) (*p* < 0.0001) compared with placebo [58]. Furthermore, in the phase III CheckMate 078 trial, an open-label, randomized study involving 504 patients with NSCLC previously treated with platinum-based doublet chemotherapy, participants were assigned a 2:1 ratio to receive either nivolumab (3 mg/kg) or docetaxel (75 mg/m²). According to Wu Y.L. and colleagues, the nivolumab group experienced a notably longer median overall survival (12 months, 95% CI: 10.4–14.0) than the 9.6 months, 95% CI: 7.6–11.2 in the docetaxel group (*p=*0.0006) [59].

This study also revealed that OS improved better in patients treated with mAb drugs than in patients who received conventional therapy. In a randomized, multi-central phase 3 trial (KEYNOTE-024 trial) involving 305 patients with advanced NSCLC (untreated previously), Reck et al. compared pembrolizumab with platinum-based chemotherapy and reported that the median overall survival (OS) was significantly greater in the pembrolizumab group (30 months) than in the platinum-based chemotherapy group (14.2 months), and the OS rates were 80.2% and 72.4% in both groups (HR=0.60, 95% CI: 0.41–0.89, *p*=0.005) [60]. Additionally, the KEYNOTE-010 trial—an international, multicenter phase II/III study—evaluated the effects of two different doses of pembrolizumab (2 mg/kg and 10 mg/kg) in comparison to docetaxel (75 mg/m²) in 1,034 patients with NSCLC who had previously progressed following platinum-based chemotherapy. Herbst R.S. and colleagues reported that the median overall survival reached 10.4 months in the low-dose pembrolizumab group, 12.7 months in the high-dose group, and 8.5 months in patients receiving docetaxel. [61]. Additionally, in another clinical study, 582 NSCLC patients were followed for 18 months. Borghaei H. et al. reported that patients treated with nivolumab survived longer than those treated with docetaxel (12.2 vs 9.4 months, respectively) [20]. Moreover, in another randomized, multicenter, open-label, phase III trial enrolling 1225 patients from community or academic oncology centers in 31 countries, Rittmeyer A. et al. revealed that the atezolizumab group had significantly longer OS than did the docetaxel group (13.8 months vs 9.6 months, respectively) [54]. Likewise, in a clinical trial including 550 individuals with locally advanced or metastatic NSCLC, Garon et al*.* demonstrated that, compared with previous therapies (10.5 months and 15.5%), pembrolizumab significantly improved both the median overall survival and 5-year survival rates in patients who had not received prior treatment (22.3 months and 23.2%, respectively) [62].

However, in the European AVAiL trial, adding bevacizumab to a cisplatin and gemcitabine regimen resulted in a statistically significant improvement in progression-free survival (*p* = 0.0003), although overall survival remained unaffected [63]. Furthermore, in a study involving 87 patients with advanced adenocarcinoma who had previously received EGFR-TKI therapy, participants were assigned to receive either a combination of osimertinib and bevacizumab or osimertinib alone. Compared with the monotherapy group, the combination group had a greater overall response rate (68%) vs (54%); however, no significant differences in progression-free survival (*p=*0.20) or overall survival (*p=*0.96) were observed between the two groups [64]. With the advancement of artificial intelligence technologies applied to multi-omics, researchers have gained deeper insights into cellular drug responses and tumor progression. These developments have opened new perspectives for precision cancer treatment, offering the potential to halt or even eliminate cancer growth and metastasis [65–67]. However, at that time, molecular stratification facilities enabling multi-omics analyses were not yet available in our region.

This study also revealed that the addition of radiation therapy to platinum-based chemotherapies improved overall survival in lung cancer patients. Based on my findings, the combination of platinum doublet therapy with an anti-PD-1/anti-PD-L1 mAb provided superior survival outcomes relative to other triple-therapy regimens, and this finding may provide guidance for determining the best therapeutic options for lung cancer patients in real-world practice and underscores the necessity of performing molecular stratification in all patients with lung cancer. The common chemotherapies used with concurrent radiation are cisplatin with etoposide or carboplatin with paclitaxel. Sause et al. reported that for patients with surgically unresectable NSCLC, combining chemotherapy with radiation provided greater survival benefits than either hyperfractionated or standard radiation therapy alone [68]. In a phase 2 clinical trial involving 276 patients with unresectable stage IIIA and IIIB NSCLC (median follow-up time of nearly 40 months), participants were randomly assigned to one of three treatment arms: a sequential arm, an induction/concurrent arm, and a concurrent/consolidation arm, paclitaxel and carboplatin used as chemotherapy, and 63 Gy of thoracic radiotherapy as radiation therapy. Belani et al. reported that overall survival was greater in the concurrent/consolidation group (16.3 months) than in the sequential and induction/concurrent groups (13.0 and 12.7 months, respectively) [69].

However, in the phase 2 SWOG-9019 trial, the addition of radiation therapy to platinum-based doublet chemotherapies [cisplatin plus etoposide with concurrent thoracic radiotherapy (TRT) regimen treatment] in fifty-one stage IIIB and 75 stage IIIA NSCLC patients did not significantly improve OS (13 and 17 months, respectively; *p=*0.810), and the three-year survival rates were 27% and 24%, respectively [70]. Furthermore, in the phase 3 PROCLAIM trial evaluating the impact of concurrent chemoradiotherapy using cisplatin with either pemetrexed or etoposide in 598 patients with unresectable stage IIIA or IIIB NSCLC, researchers did not find a significant difference in survival rates between the two groups [71].

This study demonstrated that patients who received triple therapy had significantly higher survival rates at 1, 3, and 5 years than did those who were treated with single-agent therapy or with platinum-based doublet chemotherapy, whether or not it was combined with radiation. According to a retrospective study that included 172 patients with stage IV NSCLC with a median age of 62 years (age range 41–89 years), the one-, two-, three-, and four-year survival rates were 44.2%, 21.9%, 11.6%, and 7.8%, respectively [72]. Furthermore, in a retrospective study including 846 (stage IIIA, IIIB, and IV) NSCLC patients, only 6.6% survived five years [73].

In the present study, the hazard ratio was greater in males than in females, in underweight and obese patients than in normal weight patients, in patients recognized with undifferentiated carcinoma and large cell carcinoma than in patients recognized with adenocarcinoma and squamous cell carcinoma, in smokers, in patients with chronic disease and in those who had one or more family members recognized with lung cancer. An analysis of data from 26,957 patients with NSCL revealed that improved survival was significantly and independently associated with female sex, never-smoking status, early-stage disease, squamous cell carcinoma histology, and the type of treatment received (surgery, radiation, or a combination of both) [74]

Patient age, stage at diagnosis, cancer metastasis, and undergoing lung surgery were significant predictors of overall survival in this study. In a clinical trial of 518 participants with NSCLC, the estimated five-year survival rate was 8%, and significant factors associated with reduced overall survival included male sex (HR=2.41, *p*<0.001), advanced cancer stage (HR=1.37, *p*=0.045), not receiving chemotherapy (HR=1.85, *p*=0.001), not receiving radiotherapy (HR=3.25, *p*<0.001) and cancer metastasis to the bone or liver (HR=1.44, *p*=0.009 and HR=1.57, *p*=0.016, respectively) [5]. Furthermore, in a community-based cancer study involving 20,561 patients with lung cancer, multivariate analysis revealed that male sex, age >50 years, poor performance status, advanced stage at diagnosis, and not performing lung surgery were significant independent factors associated with poor prognosis [75].

The results from this study also revealed that multiorgan metastasis was the most common type of metastasis, followed by brain, liver, and bone metastases, and patients who survived longer with lymph node, kidney, and abdominal metastases. In a retrospective study of 1,542 NSCLC patients, researchers reported that lung cancer metastasized more commonly to the bones and brain but less frequently to the liver and extrathoracic lymph nodes [3]. Moreover, in a clinical study of 518 NSCLC patients, bone and liver metastases were the predominant sites of metastasis [5], and the longest survival time was reported for patients with pleural/pericardial and contralateral lung metastases (9.03 months ± 1.41 and 8.40 months ± 1.44, respectively); however, in another clinical study of 40 lung cancer patients, metastases to the spine and limbs were the most frequently observed [4].

### Limitations

This study is subject to certain limitations inherent to its retrospective design, as the data were obtained from existing hospital records and not under controlled experimental conditions; therefore, some variables, such as patients’ symptoms and well-being, performance status, PD-L1 status, and daily cigarette consumption and length of exposure, were not recorded.

As monoclonal antibody drugs are new drugs for treating cancer, they are still expensive compared with conventional therapy and are not easily available; therefore, a limited number of patients have had the chance to be treated with mAb drugs, additionally, molecular stratification facilities were not available locally during the period to which the data pertain, which likely contributed to the limited number of patients who were eligible for, and consequently received, mAb therapy. Although potential confounding factors were documented, unmeasured or residual confounding factors, such as lifestyle factors, such as diet or physical activity, were not available in the datasets and could have influenced the outcomes.

The study sample was derived from a single oncology center (Medical Oncology Department at Hiwa Cancer Hospital), which may limit the generalizability of our findings to other populations or settings. A larger, more diverse sample would be needed to confirm these results and assess their applicability to broader patient groups.

## Conclusions

The addition of either anti-PD-1/anti-PD-L1 monoclonal, EGFRI or VEGFI to platinum-based doublet chemotherapy markedly increased the median survival and the 1-year, 3-year, and 5-year survival rates of NSCLC patients. These combinations showed a synergistic effect when taken together. These results highlight the importance of mAbs as promising therapies for lung cancer patients and the importance of performing molecular stratifications and multi-omics for all lung cancer patients. A highly statistically significant difference in overall survival was observed among the different protocols used to treat NSCLC patients. Additionally, the addition of concurrent radiation therapy to platinum-based doublet chemotherapy improved the median survival. The disease is highly metastatic, particularly to multiple organs and tissues. Patient age, disease stage at diagnosis, cancer metastasis, and lung surgical resection were significant predictors of overall survival.

## Supporting information

S1 File Protocols information. (PDF)

## Abbreviations

Anti-PD-1: Anti-programmed death 1
Anti-PD-L1: Anti-programmed death ligand 1
CPH: Cox proportional hazards
EGFRI: Epidermal growth factor inhibitors
EGFR-TKI: Epidermal growth factor receptor tyrosine kinase inhibitors
mAbs: Monoclonal antibodies
NSCLC: Non-small cell lung cancer
OS: Overall survival
PFS: Progression-free survival
SCLC: Small cell lung cancer
TAAs: Tumor-associated antigens
VEGFI: Vascular endothelial growth factor inhibitors
