# Supplementary material for: Comparison of the effects of different types of treatment protocols on the median and overall survival rates of non-small cell lung cancer patients: A real-world retrospective study
Source: PLoS One. 2026 Apr 9;21(4):e0344658. doi: 10.1371/journal.pone.0344658 (PMC13065081; doi:10.1371/journal.pone.0344658)
Supplement: S1 File — (PDF) [file pone.0344658.s001.pdf]

Treatment protocols:

Drugs	Dose & administration	Cycle
Alectinib	600 mg PO twice daily on Days 1 – 28	28-days cycle
Atezolizumab	1,200 mg IV on Day 1	21-days cycle
Bevacizumab	15 mg/kg IV on Day 1	21-days cycle
Carboplatin/Gemcitabine	Carboplatin AUC 5 IV over 30 minutes on Day 1/ Gemcitabine/1000 mg/m2 IV over 30 – 90 minutes on Days 1, 8, 15	28 days for 4-6 cycles
Carboplatin/ Paclitaxel	AUC 2 IV over 60 minutes on day1,8, 15/ Paclitaxel 80 mg/m^2 IV over 60 minutes on day1,8, 15.	21 days for 3-6 cycles
Carboplatin/Paclitaxel with Concurrent Radiation, followed by paclitaxel /carboplatin Consolidation	Carboplatin AUC 2 IV over 30 – 60 minutes on Day 1/ Paclitaxel 45 – 50 mg/m^2 IV over 60 minutes on Day 1	Weekly for 7 weeks with concurrent radiation therapy (2 cycles administered with concurrent radiation)
Carboplatin/Pemetrexed	Carboplatin AUC 5 IV over 30 minutes on Day 1/ Pemetrexed 500 mg/m^2 IV over 10 minutes on Day 1	21 days for 4-6 cycles
Carboplatin /Vinorelbine	Carboplatin AUC 6 IV over 60 minutes on Day 1/ Vinorelbine 30 mg/m^2 IV Push on Days 1 and 8/	21-days cycle for 4 cycles
Cisplatin/ Docetaxel	Cisplatin 75 mg/m2 IV over 60 minutes on Day 1/75 mg/m2 IV over 60 minutes on Day 1	21-days cycle for 6 cycles
Cisplatin/Gemcitabine	Cisplatin 75 mg/m2 IV over 60 minutes on Day 1/ Gemcitabine 1250 mg/m^2 over 30 minutes on Days 1 and 8	21-days cycle for 6 cycles
Cisplatin/Pemetrexed	Cisplatin 75 mg/m2 IV over 60 minutes on Day 1/ Pemetrexed 500 mg/m^2 IV over 10 minutes on Day 1	21-days cycle for 4 cycles (neoadjuvant or adjuvant) or 6 cycles
Cisplatin/Vinorelabine	Cisplatin 100 mg/m^2 day 1 and 8/ Vinorelbine 25 mg/m^2 day1,8,15,22,28	21 days for 4 cycles
Cisplatin/ Vinorelabine followed by Radiation	Cisplatin 100 mg/m^2 IV over 60 minutes on Days 1 and 29/ Vinorelbine 25 mg/m^2 day1,8,15,22,28	35-days cycle for 1 cycle with sequential radiation starting on Day 50
Docetaxel	75 mg/m^2 IV over 60 minutes on Day 1	21-days cycle
Erlotinib	Erlotinib 150 mg PO daily	28-days cycle
Gefitinib	250 mg Po daily on day 1 and 28	28-days cycle
Nivolumab	240 mg IV over 30 minutes on day 1	14-days cycle
Osimertinib	80 mg PO daily on Days 1 – 28	28-days cycle
Paclitaxel	260 mg/m2 IV over 30 minutes on Day 1	21-days cycle
Pembrolizumab	200 mg (or 2 mg/kg) 200 mg IV over 30 minutes on Day 1	every 6 weeks
Pemetrexed	500 mg/m^2 IV over 10 minutes on Day 1	21-days cycle
